# BABAPPAlign: a multiple sequence alignment engine with a learned residue-level scoring function

**DOI:** 10.1093/bioinformatics/btag189

**Published:** 2026-04-16

**Authors:** Krishnendu Sinha

**Affiliations:** Department of Zoology, Jhargram Raj College, Jhargram, West Bengal 721507, India

## Abstract

**Motivation:**

Multiple sequence alignment (MSA) remains a core problem in bioinformatics, yet most widely used alignment methods still rely on static amino acid substitution matrices that cannot adapt to sequence-specific context.

**Results:**

BABAPPAlign is a progressive MSA engine that replaces static substitution scoring with a trained residue-level scorer operating on fixed protein-language-model embeddings, while retaining exact affine-gap dynamic programming. It also provides an integrated codon-aware alignment mode. Using BAliBASE as the primary inferential benchmark, with supporting external validation on deterministic subsets of PREFAB and HOMSTRAD, the learned backend outperformed matched in-engine EBA-style cosine and BLOSUM62 controls, and also exceeded MAFFT.

**Availability and Implementation:**

BABAPPAlign is implemented in Python and distributed as an open-source command-line package through PyPI; the source code is available at https://github.com/sinhakrishnendu/BABAPPAlign, the archived software release is available at https://doi.org/10.5281/zenodo.17934124, and the pretrained model weights are available at https://doi.org/10.5281/zenodo.18053200.

**Supplementary material:**

Supplementary material is available at *Bioinformatics* online.

## Graphical Abstract

**Figure:**
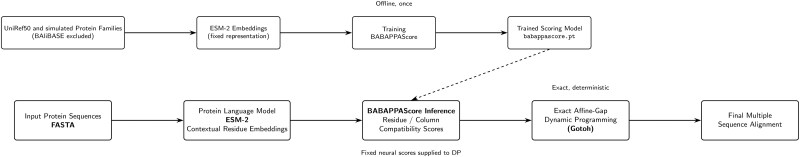

## Introduction

Multiple sequence alignment (MSA) is a foundational operation in computational biology, underpinning phylogenetic reconstruction, functional motif discovery, evolutionary rate estimation, and comparative structural analysis (Needleman and Wunsch 1970, Gotoh 1982). Despite decades of algorithmic development, accurate alignment of divergent protein families remains a central and unresolved challenge, particularly in regimes characterized by low sequence identity, compositional bias, and heterogeneous evolutionary constraints.

Most widely used MSA tools—including ClustalW, MUSCLE, MAFFT, and T-Coffee—are built upon variants of dynamic programming combined with progressive, iterative, or consistency-based heuristics (Thompson et al. 1994, Notredame et al. 2000, Edgar 2004, Katoh and Standley 2013). Although these methods differ in guide-tree construction, refinement strategies, and heuristic optimizations, they share a common reliance on static amino acid substitution matrices such as PAM and BLOSUM (Dayhoff et al. 1978, Henikoff and Henikoff 1992). These matrices encode global average substitution tendencies estimated across large and heterogeneous protein corpora and are therefore inherently context-agnostic. As a result, they are ill-suited to capture sequence-specific evolutionary pressures, structural constraints, or local biochemical environments, particularly in low-signal alignment regimes.

Recent advances in protein language models have demonstrated that rich contextual representations of amino acid residues can be learned directly from large collections of unaligned protein sequences (Rives et al. 2021, Lin et al. 2023). These models implicitly encode information about residue identity, biochemical compatibility, structural context, and long-range dependencies, suggesting that residue–residue compatibility can be inferred dynamically from sequence context rather than imposed via fixed substitution tables. While such representations have been widely adopted for structure prediction and functional annotation, their principled integration into classical sequence alignment frameworks remains limited.

One important recent example is embedding-based alignment (EBA), introduced by Pantolini et al. (2024*)*, in which residue-level protein language model embeddings are converted into a similarity matrix that is then used within a classical dynamic-programming alignment. That work established that embedding-aware score matrices can capture biologically meaningful relationships beyond those accessible to static substitution matrices. The unresolved opportunity is therefore not the use of embeddings per se, but the use of a trained residue-level scoring backend within a progressive MSA engine whose pairwise and profile–sequence alignment steps are still solved exactly by affine-gap dynamic programming, evaluated against matched in-engine controls and coupled to an integrated codon-aware alignment mode. BABAPPAlign addresses that opportunity by keeping the affine-gap dynamic-programming core fixed and replacing only the scoring layer, enabling direct comparison to both an EBA-style cosine backend and an in-engine BLOSUM62 backend.

Several recent alignment approaches have incorporated machine learning in various forms, including heuristic scoring adjustments, probabilistic models, or end-to-end differentiable alignment objectives. However, many such methods either replace classical dynamic-programming recursions with approximate inference or entangle representation learning, scoring, and optimization in ways that obscure the source of performance gains and complicate reproducibility. Consequently, it remains unclear whether reported improvements arise from improved residue compatibility modeling, altered optimization criteria, or heuristic shortcuts.

Here, BABAPPAlign is introduced as a multiple sequence alignment framework that isolates learning strictly to the residue-level scoring function while preserving the same affine-gap dynamic-programming recursion at each pairwise or profile–sequence alignment step. In BABAPPAlign, classical substitution matrices are replaced by a learned, context-aware residue compatibility function—BABAPPAScore—operating on fixed contextual embeddings derived from a pretrained protein language model. Importantly, neural inference is performed entirely outside the dynamic-programming recursion: all residue–residue or column–residue scores are computed in advance and supplied as fixed numerical inputs to the alignment algorithm. As a result, each alignment step remains exact and interpretable at the algorithmic level, and the overall procedure is fully reproducible given fixed inputs, model weights, and parameters.

This design occupies a distinct position in the MSA method landscape. By preserving the Needleman–Wunsch–Gotoh formulation and modifying only the scoring function, BABAPPAlign enables a direct assessment of how learned residue compatibility affects alignment accuracy, independent of heuristic alignment strategies or altered objective functions. The framework is architecturally modular, allowing alternative embedding backends or scoring backends to be substituted without modifying the alignment algorithm itself, although the resulting alignment quality remains backend-dependent.

In addition to protein alignment, BABAPPAlign supports codon-aware alignment of coding DNA sequences (CDS), enabling the generation of codon-consistent multiple sequence alignments suitable for downstream evolutionary analyses. Accurate alignment of CDS is essential for molecular evolutionary studies based on codon substitution models, where preservation of reading frame and codon boundaries is necessary to avoid artefacts that may bias estimates of nonsynonymous and synonymous substitution rates.

A common practice in evolutionary analysis pipelines is to align translated protein sequences and subsequently project the protein alignment back to nucleotide coordinates using external tools such as PAL2NAL (Suyama et al. 2006). While effective, this workflow separates alignment from codon-aware processing and introduces additional preprocessing steps and external dependencies. BABAPPAlign integrates this procedure directly within the alignment engine: CDS sequences are first validated, translated to protein sequences, aligned in protein space using the learned residue-level scoring framework, and then back-mapped to codon-consistent nucleotide alignments by reinserting the original codons at aligned amino-acid positions and expanding protein gaps to codon-length gaps. This approach preserves the advantages of protein-space alignment while ensuring codon boundary integrity in the resulting CDS alignment.

BABAPPAlign is evaluated using BAliBASE as the primary held-out benchmark, with supporting external validation on deterministic subsets of the PREFAB benchmark (Edgar 2010) and HOMSTRAD (Stebbings and Mizuguchi 2004). All BAliBASE reference families are excluded from training, validation, and model selection, ensuring that reported performance reflects genuine held-out evaluation. Across these benchmarks, the learned scoring backend outperforms both a matched in-engine EBA-style cosine baseline and MAFFT, while direct ablation against an in-engine BLOSUM62 backend isolates the contribution of learned scoring itself. These results establish learned residue-level scoring as a principled extension to static substitution scoring while retaining the transparency and reproducibility of classical alignment algorithms.

## Methods

### Methodological overview

**Figure 1:**
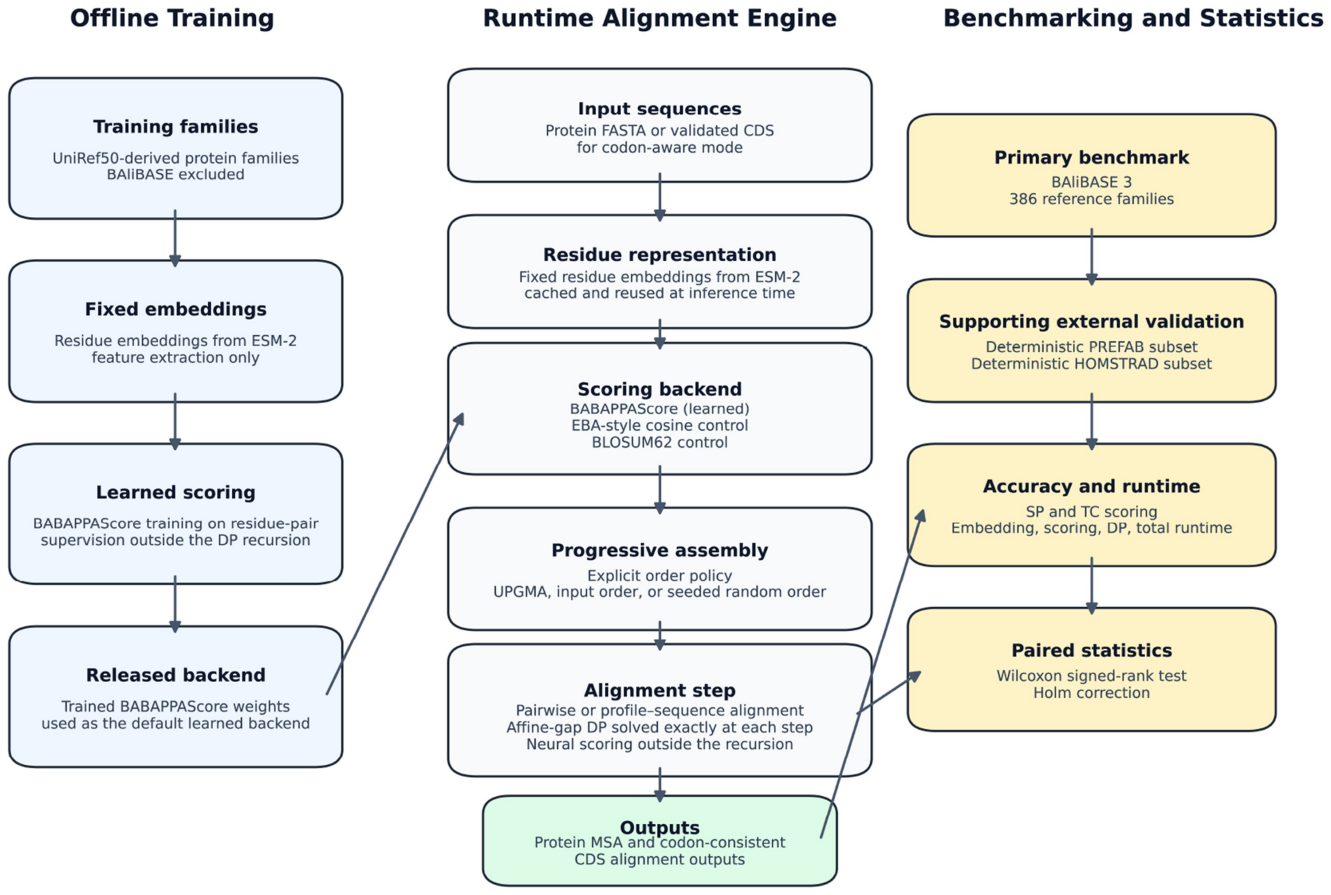
Overview of the BABAPPAlign framework. Offline training uses UniRef50-derived families with BAliBASE excluded to produce the learned BABAPPAScore backend. At runtime, BABAPPAlign combines fixed residue embeddings with one of three scoring backends—BABAPPAScore, an EBA-style cosine control, or a BLOSUM62 control—and assembles the MSA by progressive profile–sequence alignment. Each pairwise or profile–sequence alignment step is solved exactly by affine-gap dynamic programming, and BAliBASE is used as the primary benchmark with deterministic subsets from the PREFAB benchmark and HOMSTRAD as supporting external validation.

BABAPPAlign separates three components of MSA: residue representation, residue–residue scoring, and alignment optimization. Input protein sequences are first represented using fixed contextual embeddings from a pretrained protein language model. These embeddings are then converted into residue-compatibility scores using the default learned BABAPPAScore backend, or, in control experiments, by an EBA-style cosine backend or an in-engine BLOSUM62 backend. The resulting score matrix is supplied as fixed numerical input to an affine-gap dynamic-programming alignment procedure. In codon mode, CDS are validated, translated, aligned in protein space, and back-mapped to codon-consistent nucleotide alignments. Multiple alignments are assembled progressively using a defined sequence-order policy. The overall workflow is summarized in Fig. 1.

### Residue representations from protein language models

Residue-level representations were generated using a pretrained transformer-based protein language model from the ESM-2 family (esm2_t33_650M_UR50D). For a protein sequence


$$S=(s_{1},\ldots ,s_{n}),$$

The model produces contextual embeddings


$$E=(e_{1},\ldots ,e_{n}),\;e_{i}\in R^{d},$$

Where each $e_{i}$ encodes information about residue $s_{i}$ conditioned on its full sequence context. Embeddings were computed in feature-extraction mode with fixed parameters, cached for reuse, and not fine-tuned, ensuring that downstream performance differences arise from the scoring model rather than representation learning.

### BABAPPAScore: learned residue-level compatibility function

Residue compatibility in the learned mode is quantified using BABAPPAScore, the default scoring backend designed to replace static amino acid substitution matrices. For two sequences $S^{\left(A\right)}$ and $S^{\left(B\right)}$ with embeddings $E^{\left(A\right)}$ and $E^{\left(B\right)}$, compatibility between residues *i* and *j* is defined as


$$\text{Score}(i,j)=f_{\theta }\left(e_{i}^{\left(A\right)},e_{j}^{\left(B\right)}\right),$$

Where $f_{\theta }$ is a parameterized neural function. The function is explicitly symmetric,


$$f_{\theta }(x,y)=f_{\theta }(y,x),$$

ensuring consistency under sequence reordering and compatibility with classical dynamic programming frameworks.

For each sequence–sequence or profile–sequence comparison, BABAPPAScore produces a dense numerical score matrix that directly replaces classical substitution matrices, allowing residue compatibility to depend on sequence-specific contextual information.

### Progressive profile–sequence alignment

Multiple sequence alignment is performed using a progressive profile–sequence strategy in which the sequence insertion order is controlled by an explicit policy. Under the default UPGMA-based policy, a sequence-level ordering is obtained from cosine distances between sequence-embedding centroids; alternative input-order and seeded random-order policies are also supported for sensitivity analysis. This ordering affects only the sequence in which profiles are assembled and is not interpreted as a phylogenetic estimate. Given an ordered sequence list $\left(S_{1},\ldots ,S_{N}\right)$, alignment is initialized with $S_{1}$ and extended by aligning the current profile to each subsequent sequence using global affine-gap dynamic programming solved exactly for each profile–sequence alignment step.

Profile–sequence alignment follows the Needleman–Wunsch formulation with affine gap penalties as described by Gotoh (1982*)*. Let M(i,j), $I_{x}(i,j)$, and $I_{y}(i,j)$ denote the match, insertion, and deletion states. Match scores are parameterized by the selected backend evaluated between profile column embeddings and residue embeddings. All neural scoring is performed prior to dynamic programming; the alignment recursion itself operates exclusively on fixed numerical scores, yielding an exact and deterministic dynamic-programming solution for each pairwise or profile–sequence alignment instance.

### Native codon alignment mode

BABAPPAlign also has a native codon-alignment mode designed for the alignment of CDS. Codon-aware alignment is essential for downstream evolutionary analyses such as codon substitution modeling and detection of positive selection, where preservation of reading frame and codon boundaries is required.

When codon mode is enabled, input nucleotide sequences are first validated to ensure biological consistency. Each CDS must satisfy three conditions: (i) sequence length divisible by three, (ii) absence of internal stop codons, and (iii) conformity to the canonical nucleotide alphabet. Sequences failing these checks are rejected to prevent frame-disrupting alignments.

Following validation, CDS sequences are translated into amino acid sequences using the standard genetic code. Alignment is then performed entirely in protein space using the BABAPPAlign framework described above, including contextual residue embeddings and the learned residue-level compatibility scoring model. This protein-first strategy leverages the greater evolutionary signal present at the amino acid level while preserving the mapping back to the original codons.

After protein alignment is completed, the aligned amino acid sequences are back-mapped to nucleotide coordinates to produce an aligned CDS file. Each non-gap amino acid position in the aligned protein is replaced by its original codon from the input CDS, whereas each gap in the protein alignment is expanded to a codon-length gap in the nucleotide alignment. The final nucleotide alignment therefore follows the topology of the aligned protein sequences while preserving codon boundaries. This back-mapping procedure is conceptually similar to the PAL2NAL strategy but is implemented internally within BABAPPAlign, eliminating the need for external post-processing tools.

Gap penalties in codon mode are automatically scaled to maintain biological consistency with codon-length insertions and deletions. Importantly, the underlying alignment optimization remains unchanged: dynamic programming is performed in protein space using the same affine-gap recursion solved exactly for each alignment step, and neural scoring is computed outside the alignment recursion as in the standard protein mode. This design preserves the methodological advantages of learned residue-level scoring and alignment-step exactness in the codon-alignment context, while providing a seamless and integrated solution for codon-consistent multiple sequence alignment.

### Training data and benchmark isolation

Training data for BABAPPAScore were derived from curated protein families obtained from UniRef50. All BAliBASE reference families were strictly excluded from training, validation, and model selection to prevent information leakage. BAliBASE therefore served as the primary held-out family-level benchmark in the revised evaluation. The PREFAB benchmark and HOMSTRAD were used only for supporting external validation under fixed model and parameter settings.

Protein families were processed into standardized FASTA format and organized into deterministic shards to enable scalable preprocessing and exact reproducibility.

### Benchmarking protocol

Benchmarking was conducted using a dedicated reproducible pipeline. BAliBASE 3 (RV11–RV50) (Thompson et al. 1999) served as the primary inferential benchmark and was evaluated in full across all 386 reference families. Supporting external validation used a deterministic 250-case subset of the PREFAB benchmark (Edgar 2010) and a deterministic 100-family subset of HOMSTRAD (Stebbings and Mizuguchi 2004). Deterministic subsetting on the external datasets was used to provide reproducible secondary validation and runtime characterization at tractable computational cost under fixed model and parameter settings; no central claim in the revised manuscript depends solely on those external subsets.

The primary head-to-head comparison used three methods under a shared evaluation framework: BABAPPAlign with the learned scorer, a matched in-engine EBA-style cosine backend, and MAFFT. Additional experiment blocks evaluated (i) sensitivity to varied affine-gap penalties on a stratified 60-family BAliBASE subset, (ii) matched in-engine ablation of the learned scorer against an in-engine BLOSUM62 backend, (iii) backend-swappability across ESM-2 and ESM-1b under fixed alignment logic, and (iv) an indicative sensitivity analysis of UPGMA, input-order, and seeded random-order progressive policies on a focused six-family BAliBASE subset.

Alignment accuracy was assessed using a single scoring implementation to compute sum-of-pairs (SP) and total column (TC) scores for all benchmarks. Runtime was profiled separately for embedding computation, scoring, dynamic programming, and total wall-clock time. Benchmarking was performed serially to eliminate nondeterminism. Execution logs, runtimes, and failure modes were recorded for each family and method.

### Statistical analysis and reproducibility

All comparisons were conducted as paired, family-wise analyses. Differences in SP and TC were evaluated using the Wilcoxon signed-rank test (Wilcoxon 1945), and Holm correction was applied across pairwise method comparisons within each experiment and metric (Holm 1979). The study reports mean SP/TC values for each method, median paired deltas for direct contrasts, and median/IQR summaries for runtime phases. Gap-robustness analyses were summarized as median and median absolute shifts relative to the baseline affine-gap setting.

BABAPPAlign is deterministic given fixed inputs, model weights, and parameters. Residue embeddings were cached, scoring models were versioned, and alignment parameters were held constant. Experiments were conducted on Linux systems; GPU acceleration affected performance only, not correctness. Under identical conditions, BABAPPAlign produces identical alignments across runs.

## Results

### Primary BAliBASE comparison with supporting external validation

BAliBASE served as the primary inferential benchmark and was evaluated across all 386 reference families using paired family-wise comparisons. Supporting external validation used deterministic subsets of the PREFAB benchmark and HOMSTRAD to assess whether the same method ranking was reproduced on additional benchmark collections under fixed model and parameter settings.

On BAliBASE, the learned backend produced the highest mean SP and TC values (Table 1), achieving 0.7580/0.3780 compared with 0.7175/0.3512 for MAFFT and 0.5783/0.2097 for the matched in-engine EBA-style cosine control. The same ranking was reproduced on the supporting external validation sets. On PREFAB, learned scoring achieved mean SP/TC of 0.6132/0.6143, compared with 0.5462/0.5597 for MAFFT and 0.4666/0.4887 for the EBA-style cosine backend. On HOMSTRAD-100, learned scoring reached 0.8876/0.6809 versus 0.8310/0.6218 for MAFFT and 0.7056/0.4411 for the EBA-style cosine backend.

**Table 1:** Primary benchmark summary for BAliBASE and supporting external validation sets, comparing BABAPPAlign with learned scoring, the matched in-engine EBA-style cosine backend, and MAFFT.

Dataset	Learned	MAFFT	EBA-cosine
BAliBASE (n=386)	0.7580/0.3780	0.7175/0.3512	0.5783/0.2097
PREFAB (n=250)	0.6132/0.6143	0.5462/0.5597	0.4666/0.4887
HOMSTRAD-100 (n=100)	0.8876/0.6809	0.8310/0.6218	0.7056/0.4411

Values are reported as mean SP/mean TC.

Paired Wilcoxon signed-rank tests with Holm correction showed that these gains were statistically significant in all principal contrasts. On BAliBASE, learned versus MAFFT produced median SP and TC deltas of +0.0217 and +0.0218, with Holm-adjusted *p* values of $1.07\times 10^{-19}$ and $3.52\times 10^{-12}$, respectively. Learned versus the EBA-style cosine baseline produced markedly larger BAliBASE median deltas of +0.1615 (SP) and +0.1554 (TC), with Holm-adjusted *p* values of $1.16\times 10^{-56}$ and $3.05\times 10^{-53}$. On PREFAB, learned versus MAFFT produced median SP and TC deltas of +0.0429 and +0.0379, whereas learned versus the EBA-style cosine baseline produced median deltas of +0.0911 and +0.0742; all corresponding Holm-adjusted *p* values remained below $10^{-13}$. The supplementary HOMSTRAD-100 benchmark showed the same direction of effect, with learned versus MAFFT median deltas of +0.0268 (SP) and +0.0429 (TC), and learned versus EBA-style cosine median deltas of +0.1404 (SP) and +0.2011 (TC), all significant after Holm correction.

These results establish that the strongest head-to-head comparison is not simply against classical aligners, but also against an explicit embedding-based dynamic-programming baseline. In the completed benchmarks, the learned scorer is consistently superior to both MAFFT and the matched in-engine EBA-style cosine backend. For completeness, the earlier BAliBASE-only comparisons against additional classical aligners (ClustalW, MUSCLE, and T-Coffee) are retained in the Supplementary Materials, available as supplementary data at *Bioinformatics* online as secondary baseline context.

### Gap robustness and learned-scoring ablation

To test robustness to gap penalties, the learned scorer was evaluated on a stratified 60-family BAliBASE subset over a 3×3 affine-gap grid with gap-open values of −3.5, −2.5, and −1.5 and gap-extend values of −1.0, −0.7, and −0.3. Relative to the baseline setting (−2.5, −0.7), the maximum median absolute shift was 0.0066 for SP and 0.0125 for TC, indicating that the learned method is stable to moderate variation in affine-gap settings.

To isolate the contribution of the learned scoring function, the study replaced the learned scorer with an in-engine BLOSUM62 backend while keeping the progressive affine-gap optimizer fixed. This ablation showed that the observed gains are primarily attributable to the learned scoring model rather than to the dynamic-programming core. On BAliBASE, learned scoring achieved 0.7580/0.3780 compared with 0.3537/0.0873 for BLOSUM62, with median learned-minus-BLOSUM62 deltas of +0.4109 (SP) and +0.2891 (TC) and Holm-adjusted *p* values of $5.42\times 10^{-65}$ and $2.52\times 10^{-63}$. The same pattern held on PREFAB and on the supplementary HOMSTRAD-100 benchmark (Table 2).

**Table 2:** Learned-versus-BLOSUM62 ablation under the same progressive affine-gap dynamic-programming core.

Dataset	Learned	BLOSUM62	Holm *p* (SP)	Holm *p* (TC)
BAliBASE (n=386)	0.7580/0.3780	0.3537/0.0873	$5.42\times 10^{-65}$	$2.52\times 10^{-63}$
PREFAB (n=250)	0.6132/0.6143	0.2514/0.3141	$2.67\times 10^{-40}$	$4.23\times 10^{-40}$
HOMSTRAD-100 (n=100)	0.8876/0.6809	0.5504/0.3127	$3.90\times 10^{-18}$	$4.96\times 10^{-18}$

### Backend-Swappability, runtime, and order sensitivity

The completed ESM-2/ESM-1b experiment should be interpreted as a backend-swappability analysis rather than as evidence of backend-invariant performance. The alignment engine remained unchanged across these runs, demonstrating architectural modularity. However, performance remained strongly backend-dependent: in the present zero-shot setting, ESM-2 consistently outperformed ESM-1b for both the learned backend and the EBA-style cosine backend across BAliBASE, PREFAB, and HOMSTRAD (Table 3). Thus, the framework is backend-swappable, but ESM-2 is the preferred default on current evidence.

**Table 3:** Zero-shot embedding-backend swappability experiment. Values are reported as mean SP/mean TC.

Dataset	Learned ESM-2	Learned ESM-1b	EBA-cosine ESM-2	EBA-cosine ESM-1b
BAliBASE (n=386)	0.7580/0.3780	0.3829/0.1201	0.5783/0.2097	0.3760/0.1147
PREFAB (n=250)	0.6132/0.6143	0.3254/0.3748	0.4666/0.4887	0.3026/0.3568
HOMSTRAD-100 (n=100)	0.8876/0.6809	0.5265/0.2557	0.7056/0.4411	0.5165/0.2449

The phase-level runtime profiles clarify where the computational trade-off arises. The dominant additional cost of the learned mode lies in scoring rather than in the dynamic-programming recursion itself (Table 4). On BAliBASE, median DP times were 6.14 s for learned scoring and 5.39 s for EBA-style cosine in the primary R1 comparison, whereas median scoring times were 34.37 s and 0.15 s, respectively. On the BAliBASE learned-versus-BLOSUM62 ablation, median DP times remained comparable (6.13 s versus 6.55 s), but median scoring times differed strongly (33.65 s versus 7.35 s). A similar pattern held on HOMSTRAD-100, where learned scoring substantially exceeded EBA-style cosine in embedding and scoring time while preserving similar DP costs.

**Table 4:** Representative median runtime decomposition (seconds) for embedding, scoring, and dynamic programming.

Experiment	Method	Embedding	Scoring	DP
BAliBASE R1	Learned	0.0741	34.3708	6.1363
BAliBASE R1	EBA-cosine	0.0141	0.1536	5.3924
BAliBASE ablation	Learned	0.0840	33.6484	6.1276
BAliBASE ablation	BLOSUM62	0.0109	7.3508	6.5523
HOMSTRAD-100 R1	Learned	4.1240	2.4236	0.4194
HOMSTRAD-100 R1	EBA-cosine	0.0030	0.0143	0.3896

As an indicative sensitivity analysis, the study evaluated six stratified BAliBASE families under UPGMA, input-order, and seeded random-order progressive policies. Mean SP/TC values were 0.6593/0.2265 for UPGMA, 0.6571/0.2508 for input order, and 0.6658/0.2712 for random order; no pairwise contrast remained significant after Holm correction. These results suggest limited order sensitivity in this focused setting, while still leaving broader progressive-order effects as an appropriate topic for future study.

## Discussion and conclusion

The revised analyses support a narrow but strong conclusion. BABAPPAlign is not the first method to combine contextual residue embeddings with classical dynamic programming; rather, its contribution is to implement a progressive MSA engine in which the pairwise and profile–sequence alignment steps continue to use the same affine-gap dynamic-programming recursion, while the scoring layer alone is replaced by a trained residue-level backend. Within that fixed optimization framework, the learned scorer outperformed a matched EBA-style cosine control, a matched BLOSUM62 control, and MAFFT on BAliBASE, with supporting external validation on deterministic subsets of the PREFAB benchmark and HOMSTRAD.

The clearest mechanistic result is the matched in-engine BLOSUM62 ablation. Replacing the learned scorer with BLOSUM62 within the same alignment engine caused large and highly significant drops in both SP and TC on BAliBASE, PREFAB, and HOMSTRAD-100. This shows that the main observed gain is attributable to learned, context-aware residue compatibility rather than to a change in optimization strategy.

The backend-swappability analyses also sharpen the interpretation of the machine-learning component. The architecture supports substitution of alternative embedding backends without changing the affine-gap dynamic-programming core, but the completed experiments show that alignment quality remains backend-dependent. In the present zero-shot setting, ESM-2 was consistently stronger than ESM-1b for both the learned backend and the EBA-style cosine backend.

Phase-resolved runtime analyses reveal a similarly clear pattern. The principal computational premium of the learned method lies in embedding and scoring, whereas the dynamic-programming phase remains in the same general range across backends. This identifies a concrete engineering target for future optimization without challenging the current alignment-step dynamic-programming formulation.

### Relevance for codon-based evolutionary analyses

Accurate codon-aware multiple sequence alignments are a critical prerequisite for evolutionary analyses based on codon substitution models, including estimation of nonsynonymous to synonymous substitution rates ($d_{N}/d_{S}$) and detection of episodic positive selection. In such analyses, frame-preserving alignment and codon boundary integrity are essential to avoid spurious signals of selection.

The native codon-alignment mode implemented in BABAPPAlign enables the direct generation of codon-consistent multiple sequence alignments by validating CDS, translating them to protein sequences, aligning those proteins, and then back-translating the aligned proteins to nucleotide space using the original codons. This strategy combines the improved evolutionary signal available at the amino acid level with strict preservation of codon structure in the resulting nucleotide alignment.

Consequently, alignments generated by BABAPPAlign can be used directly in downstream codon-based evolutionary frameworks such as PAML, HyPhy, and related likelihood-based phylogenetic inference tools without requiring external post-processing steps. This capability simplifies evolutionary analysis pipelines and reduces the risk of frame-disrupting alignment artifacts that can influence inference of selection.

Several limitations should be stated explicitly. Progressive alignment remains order-sensitive in principle, and the present order analysis is an indicative sensitivity test on a small stratified subset rather than a general proof of order invariance. The external PREFAB and HOMSTRAD analyses are supporting validation sets rather than the primary inferential basis of the manuscript. Performance also remains backend-dependent: the architecture supports substitution of alternative embedding backends, but zero-shot ESM-1b was consistently weaker than ESM-2 in the present experiments. In addition, the learned backend incurs a runtime premium, with most additional cost arising from embedding and scoring rather than from the affine-gap dynamic-programming recursion. Finally, the nucleotide functionality is an integrated codon-aware alignment mode implemented through protein-space alignment and back-mapping; extension to non-coding nucleotide alignment would require different training targets and scoring models.

Looking forward, the separation of representation, scoring, and optimization introduced here opens several concrete directions for future work. Alternative protein language models, lighter-weight embedding architectures, or domain-specific representations can be evaluated within the same alignment core. The scoring layer could also be extended to profile–profile alignment while retaining the same affine-gap dynamic-programming solution at each alignment step. More broadly, the present results support further investigation of learned, context-aware scoring backends within classical alignment frameworks.

In conclusion, BABAPPAlign provides a progressive multiple sequence alignment engine in which each pairwise or profile–sequence alignment step is solved exactly by affine-gap dynamic programming, while static substitution scoring is replaced by a trained residue-level backend. The completed BAliBASE analyses, together with supporting external validation on deterministic subsets of the PREFAB benchmark and HOMSTRAD, show that this learned-scoring formulation outperforms a matched EBA-style cosine control, a matched BLOSUM62 control, and MAFFT under paired family-wise evaluation. These results support learned scoring as a technically defensible extension of classical alignment methodology rather than a replacement for the underlying optimization framework.

## Implementation and availability

BABAPPAlign is implemented in Python and distributed as an open-source command-line package through PyPI (pip install babappalign). Source code is available at https://github.com/sinhakrishnendu/BABAPPAlign, and an archived software release is available via Zenodo at https://doi.org/10.5281/zenodo.17934124. The pretrained BABAPPAScore model weights are distributed separately via Zenodo at https://doi.org/10.5281/zenodo.18053200 and are specified explicitly at runtime. The repository contains the benchmarking scripts and result tables used for the revised analyses. Under fixed input FASTA files, model weights, and parameter settings, BABAPPAlign is deterministic; GPU acceleration affects runtime only and not the computed alignments.

## Supplementary Material

btag189_Supplementary_Data

## Data Availability

The data underlying this article are available in https://doi.org/10.5281/zenodo.19439608.
