# Supplementary material for: BABAPPAlign: a multiple sequence alignment engine with a learned residue-level scoring function
Source: Bioinformatics. 2026 Apr 16;42(5):btag189. doi: 10.1093/bioinformatics/btag189 (PMC13141150; doi:10.1093/bioinformatics/btag189)
Supplement: btag189_Supplementary_Data [file btag189_supplementary_data.pdf]

Supplementary Material for BABAPPAlign

Krishnendu Sinha*

Supplementary Benchmark and Sensitivity Analyses

This Supplementary Material provides benchmark, control, and sensitivity analyses supporting the revised evaluation of BABAPPAlign. Sections 1–6 summarize the primary benchmark framework used in the revised manuscript, including comparison to a matched embedding-similarity control, matched in-engine ablation of the learned scoring backend, sensitivity to affine-gap parameters, backend-swappability across embedding models, phase-resolved runtime decomposition, and an indicative progressive-order sensitivity analysis. Sections 7 and 8 provide additional BAliBASE comparisons against classical aligners as supplementary context.

1. Primary Comparison with a Matched Embedding-Similarity Control and MAFFT

The primary revised comparison evaluated BABAPPAlign with learned scoring against two comparators under a shared evaluation framework: a matched in-engine embedding-similarity control (EBA-style cosine scoring) and MAFFT. BAliBASE served as the primary inferential benchmark and was evaluated in full across 386 reference families. Deterministic subsets of the PREFAB benchmark of Edgar (2010) and HOMSTRAD were used as supporting external validation sets under fixed model and parameter settings.

Across all three datasets, the learned backend ranked first in both SP and TC. Paired Wilcoxon signed-rank tests with Holm correction showed that the principal contrasts were significant throughout the primary comparison framework:

- BAliBASE learned versus MAFFT: median SP delta = +0.0217, Holm-adjusted $p = 1.07 \times 10^{-19}$; median TC delta = +0.0218, Holm-adjusted $p = 3.52 \times 10^{-12}$.
- BAliBASE learned versus EBA-cosine: median SP delta = +0.1615, Holm-adjusted $p = 1.16 \times 10^{-56}$; median TC delta = +0.1554, Holm-adjusted $p = 3.05 \times 10^{-53}$.
- PREFAB learned versus MAFFT: median SP delta = +0.0429, Holm-adjusted $p = 7.62 \times 10^{-16}$; median TC delta = +0.0379, Holm-adjusted $p = 3.79 \times 10^{-14}$.
- PREFAB learned versus EBA-cosine: median SP delta = +0.0911, Holm-adjusted $p = 1.34 \times 10^{-21}$; median TC delta = +0.0742, Holm-adjusted $p = 9.13 \times 10^{-20}$.

*Corresponding author: dr.krishnendusinha@gmail.com

Table 1: Primary paired family-wise comparison of BABAPPAlign with learned scoring, the matched in-engine EBA-style cosine backend, and MAFFT on BAliBASE and on deterministic supporting external validation subsets from the PREFAB benchmark of Edgar (2010) and HOMSTRAD. Values are reported as mean SP and mean TC.

Dataset	Method	Mean SP	Mean TC	n
BAliBASE	BABAPPAlign learned	0.7580	0.3780	386
BAliBASE	MAFFT	0.7175	0.3512	386
BAliBASE	BABAPPAlign EBA-cosine	0.5783	0.2097	386
PREFAB	BABAPPAlign learned	0.6132	0.6143	250
PREFAB	MAFFT	0.5462	0.5597	250
PREFAB	BABAPPAlign EBA-cosine	0.4666	0.4887	250
HOMSTRAD-100	BABAPPAlign learned	0.8876	0.6809	100
HOMSTRAD-100	MAFFT	0.8310	0.6218	100
HOMSTRAD-100	BABAPPAlign EBA-cosine	0.7056	0.4411	100

- HOMSTRAD-100 learned versus MAFFT: median SP delta = +0.0268, Holm-adjusted $p = 4.08 \times 10^{-8}$; median TC delta = +0.0429, Holm-adjusted $p = 7.86 \times 10^{-6}$.
- HOMSTRAD-100 learned versus EBA-cosine: median SP delta = +0.1404, Holm-adjusted $p = 6.47 \times 10^{-17}$; median TC delta = +0.2011, Holm-adjusted $p = 9.60 \times 10^{-17}$.

The principal inferential conclusion is therefore based on BAliBASE and supported by consistent ranking on the external validation subsets: within the revised evaluation framework, the learned scoring backend outperformed both the matched embedding-similarity control and MAFFT.

2. Sensitivity to Affine-Gap Parameters

Sensitivity to affine-gap penalties was assessed on a stratified 60-family BAliBASE subset spanning RV11, RV12, RV20, RV30, RV40, and RV50. Each family was evaluated across a 3×3 grid of gap-open and gap-extend values.

Table 2: Sensitivity of BABAPPAlign with learned scoring to affine-gap settings on a stratified 60-family BAliBASE subset. Values are summarized relative to the baseline setting `gap-open=-2.5` and `gap-extend=-0.7`.

Gap open	Gap extend	n cases	Median $\lvert\Delta\text{SP}\rvert$	Median $\lvert\Delta\text{TC}\rvert$
−3.5	−1.0	60	0.0037	0.0068
−3.5	−0.7	60	0.0042	0.0060
−3.5	−0.3	60	0.0058	0.0101
−2.5	−1.0	60	0.0031	0.0028
−2.5	−0.7	60	0.0000	0.0000
−2.5	−0.3	60	0.0033	0.0060
−1.5	−1.0	60	0.0066	0.0125
−1.5	−0.7	60	0.0051	0.0122
−1.5	−0.3	60	0.0041	0.0090

The largest median absolute shifts were 0.0066 for SP and 0.0125 for TC, indicating limited variation across moderate affine-gap perturbations on this benchmark subset.

3. Matched In-Engine Ablation of the Learned Scoring Backend

To isolate the contribution of the learned scoring layer, the learned backend was replaced by an in-engine BLOSUM62 backend while keeping the progressive affine-gap dynamic-programming core fixed. This matched-control design isolates the effect of the scoring function from the effect of the optimizer.

Table 3: Matched in-engine ablation of the learned scoring backend against a BLOSUM62 backend across the completed benchmark collections. Values are reported as mean SP and mean TC; Holm-adjusted p values refer to paired Wilcoxon contrasts between learned scoring and BLOSUM62.

Dataset	Learned (SP/TC)	BLOSUM62 (SP/TC)	Holm p (SP)	Holm p (TC)
BAliBASE ($n = 386$)	0.7580 / 0.3780	0.3537 / 0.0873	5.42×10^{-65}	2.52×10^{-63}
PREFAB ($n = 250$)	0.6132 / 0.6143	0.2514 / 0.3141	2.67×10^{-40}	4.23×10^{-40}
HOMSTRAD-100 ($n = 100$)	0.8876 / 0.6809	0.5504 / 0.3127	3.90×10^{-18}	4.96×10^{-18}

This matched in-engine ablation provides the clearest mechanistic result in the revised evaluation. Across BAliBASE, PREFAB, and HOMSTRAD-100, the learned scorer outperformed the BLOSUM62 backend under the same affine-gap dynamic-programming core.

4. Backend-Swappability Across Embedding Models

Backend-swappability was evaluated by substituting ESM-2 and ESM-1b embeddings while leaving the alignment engine unchanged. This analysis addresses architectural modularity rather than backend-invariant performance.

Table 4: Backend-swappability analysis across ESM-2 and ESM-1b for both the learned and EBA-style cosine backends. Values are reported as mean SP and mean TC.

Dataset	Learned ESM-2	Learned ESM-1b	EBA-cosine ESM-2	EBA-cosine ESM-1b
BAliBASE ($n = 386$)	0.7580 / 0.3780	0.3829 / 0.1201	0.5783 / 0.2097	0.3760 / 0.1147
PREFAB ($n = 250$)	0.6132 / 0.6143	0.3254 / 0.3748	0.4666 / 0.4887	0.3026 / 0.3568
HOMSTRAD-100 ($n = 100$)	0.8876 / 0.6809	0.5265 / 0.2557	0.7056 / 0.4411	0.5165 / 0.2449

The implementation is backend-swappable, but performance remained backend-dependent in the present zero-shot setting. ESM-2 was consistently stronger than ESM-1b for both the learned backend and the EBA-style cosine backend across all three benchmark collections.

5. Phase-Resolved Runtime Decomposition

Runtime was decomposed into embedding, scoring, and dynamic-programming phases. The purpose of this analysis was to identify where the computational premium of learned scoring arises.

Table 5: Representative median phase timings (seconds) for BABAPPAlign and matched controls. Values are medians over families within each experiment.

Experiment	Method	Embedding	Scoring	DP
BAliBASE R1	Learned	0.0741	34.3708	6.1363
BAliBASE R1	EBA-cosine	0.0141	0.1536	5.3924
BAliBASE ablation	Learned	0.0840	33.6484	6.1276
BAliBASE ablation	BLOSUM62	0.0109	7.3508	6.5523
HOMSTRAD-100 R1	Learned	4.1240	2.4236	0.4194
HOMSTRAD-100 R1	EBA-cosine	0.0030	0.0143	0.3896

Across experiments, the dominant additional cost of the learned backend was concentrated in embedding and scoring, whereas dynamic-programming times remained in a similar range across backends.

6. Indicative Progressive-Order Sensitivity Analysis

An indicative sensitivity analysis evaluated six stratified BAliBASE families under UPGMA, input-order, and seeded random-order progressive policies.

Table 6: Indicative progressive-order sensitivity analysis on a six-family stratified BAliBASE subset. Values are reported as mean SP and mean TC.

Order mode	Mean SP	Mean TC	n
UPGMA	0.6593	0.2265	6
Input order	0.6571	0.2508	6
Random order	0.6658	0.2712	6

No pairwise differences were Holm-significant in this focused experiment. The result therefore supports only a modest conclusion: order effects were limited in this small sensitivity analysis, but the experiment does not establish general order invariance for progressive alignment.

Additional BAliBASE Classical Baseline Analyses

Additional BAliBASE classical-baseline analyses are provided here as supplementary context. These summaries use the original effect-size, confidence-interval, and FDR-adjusted statistics from that BAliBASE comparison and are supplementary to the primary benchmark framework reported in the main manuscript.

7. Overall BAliBASE Comparison with Classical Aligners

In this supplementary BAliBASE classical-baseline analysis, BABAPPAlign was compared against four classical aligners: ClustalW, MUSCLE, MAFFT, and T-Coffee. All 386 BAliBASE reference families were evaluated under a paired design in which BABAPPAlign and each comparator were run on the same unaligned inputs.

Table 7: Additional BAliBASE paired family-wise comparison between BABAPPAlign and four classical aligners. Positive score differences indicate higher SP or TC for BABAPPAlign; positive runtime differences indicate longer runtime for BABAPPAlign.

Metric	Comparator	Δ Median	95% CI	Cliff's δ	FDR p
SP	ClustalW	0.0669	[0.0547, 0.0837]	0.751	2.7×10^{-48}
SP	MAFFT	0.0252	[0.0190, 0.0306]	0.523	3.2×10^{-29}
SP	MUSCLE	0.0512	[0.0419, 0.0664]	0.626	8.7×10^{-40}
SP	T-Coffee	0.0642	[0.0536, 0.0782]	0.668	5.2×10^{-46}
TC	ClustalW	0.0665	[0.0534, 0.0785]	0.491	2.1×10^{-27}
TC	MAFFT	0.0255	[0.0187, 0.0316]	0.393	8.7×10^{-18}
TC	MUSCLE	0.0183	[0.0115, 0.0236]	0.279	4.2×10^{-10}
TC	T-Coffee	0.0382	[0.0292, 0.0470]	0.495	1.2×10^{-22}

In this BAliBASE-only baseline analysis, BABAPPAlign outperformed all four classical aligners. The largest median SP advantage was observed against ClustalW, followed by T-Coffee and MUSCLE, whereas MAFFT was the strongest comparator among the classical tools included in that evaluation.

8. BAliBASE Performance by Reference Variability Category

The BAliBASE classical-baseline analysis also stratified families by reference variability (RV) category to examine whether the relative advantage of BABAPPAlign depended on alignment difficulty. These RV categories range from low variability (RV11) to highly variable and ambiguous reference alignments (RV50).

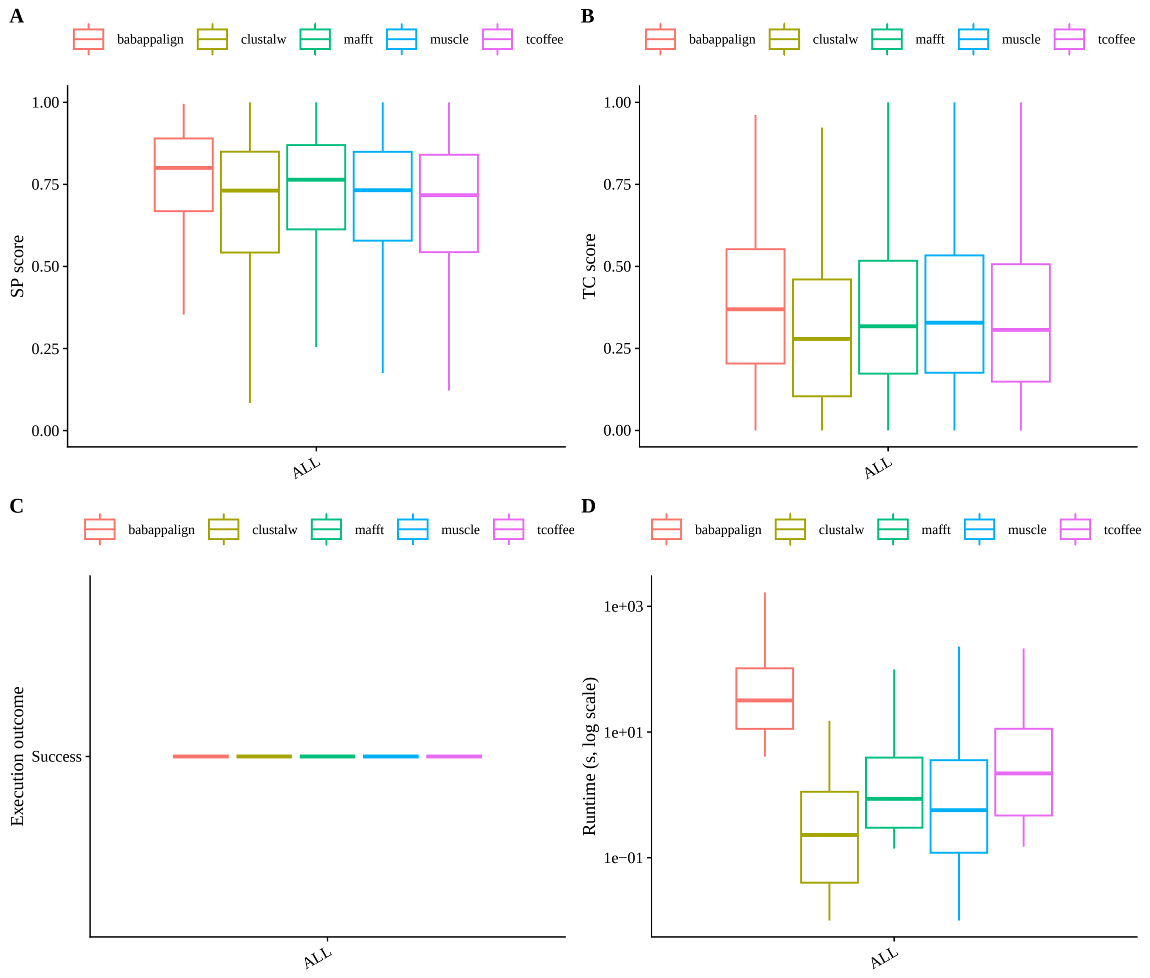

Figure 1: Additional BAliBASE paired family-wise comparison between BABAPPAlign and four classical aligners. Boxplots show paired differences (BABAPPAlign minus comparator) in SP score, TC score, and runtime across 386 BAliBASE families. Positive score differences indicate higher alignment accuracy for BABAPPAlign; positive runtime differences indicate longer runtime for BABAPPAlign.

Table 8: Additional BAliBASE paired family-wise comparison between BABAPPAlign and classical aligners, stratified by reference variability category. Positive Δ values indicate higher SP, TC, or runtime for BABAPPAlign relative to the corresponding comparator summary within each RV class.

RV	Metric	n	Δ Median	95% CI	Cliff's δ	FDR p
RV11	SP	76	0.2096	[0.1603, 0.2177]	0.8158	2.6×10^{-11}
	TC	76	0.1405	[0.1154, 0.1753]	0.8133	1.8×10^{-10}
	Runtime (s)	76	8.61	[7.36, 10.64]	1.000	3.7×10^{-14}
RV12	SP	88	0.0420	[0.0337, 0.0573]	0.6136	1.6×10^{-10}
	TC	88	0.0530	[0.0235, 0.0789]	0.4091	1.8×10^{-6}
	Runtime (s)	88	15.16	[10.84, 21.63]	1.000	3.8×10^{-16}

RV	Metric	n	Δ Median	95% CI	Cliff's δ	FDR p
RV20	SP	82	0.0855	[0.0642, 0.1173]	0.8049	7.1×10^{-13}
	TC	82	0.0919	[0.0740, 0.1107]	0.5802	5.5×10^{-8}
	Runtime (s)	82	55.38	[39.31, 86.90]	1.000	3.7×10^{-15}
RV30	SP	60	0.0861	[0.0489, 0.1029]	0.8000	2.7×10^{-8}
	TC	60	0.0671	[0.0483, 0.0898]	0.6207	2.1×10^{-5}
	Runtime (s)	60	97.16	[59.45, 117.09]	1.000	1.7×10^{-11}
RV40	SP	49	0.0604	[0.0450, 0.0714]	0.7551	8.8×10^{-7}
	TC	49	0.0165	[0.0010, 0.0356]	0.3333	2.0×10^{-1}
	Runtime (s)	49	92.42	[64.27, 136.07]	1.000	1.1×10^{-9}
RV50	SP	31	0.1128	[0.0507, 0.1360]	0.8710	6.6×10^{-6}
	TC	31	0.0377	[0.0177, 0.0713]	0.4839	7.6×10^{-4}
	Runtime (s)	31	74.22	[36.75, 141.41]	1.000	1.2×10^{-6}

Within this stratified BAliBASE analysis, the largest gains were observed in RV20 and RV30, whereas RV40 and RV50 remained positive but showed weaker TC-based significance. These summaries provide additional baseline context alongside the primary benchmark framework reported in the main manuscript.

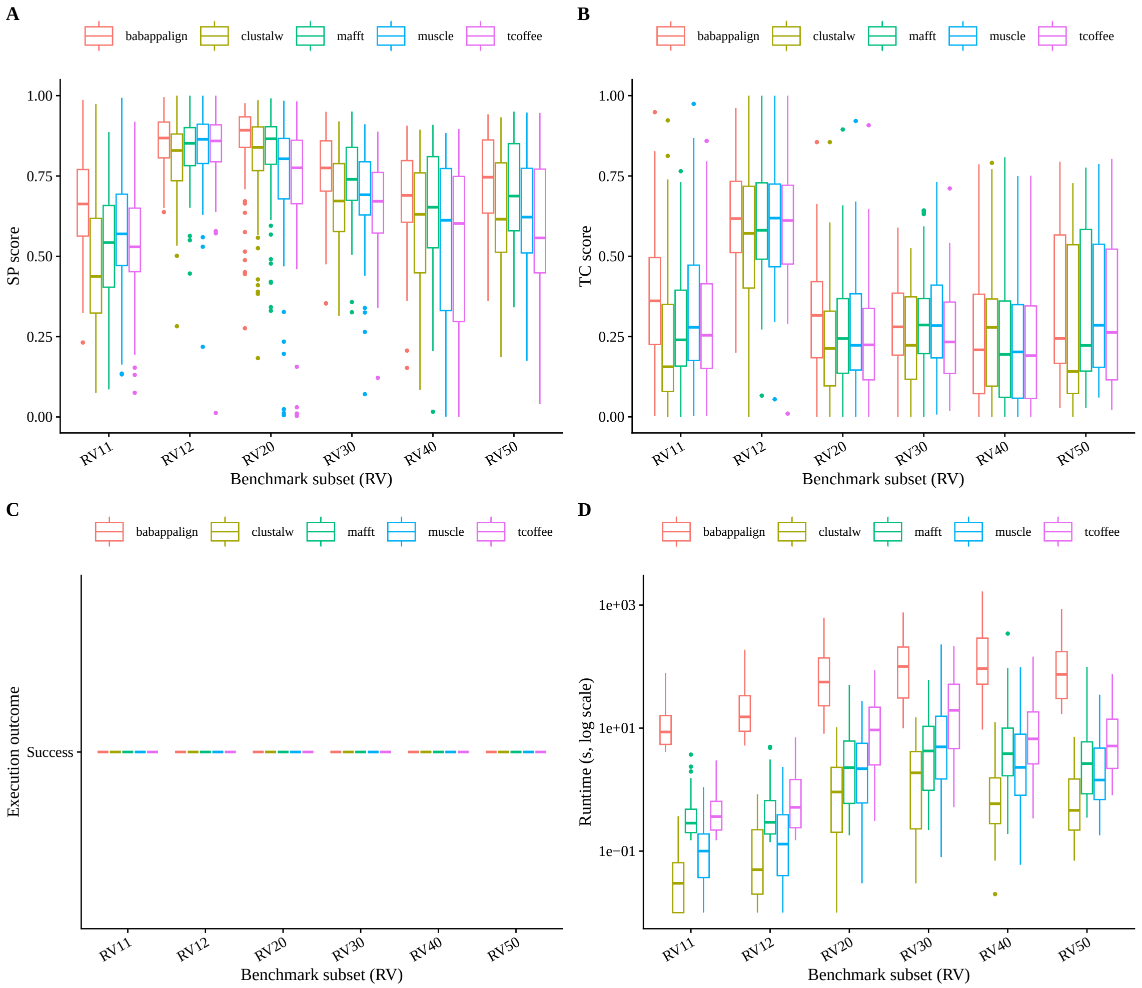

Figure 2: Additional BAliBASE paired family-wise comparison between BABAPPAlign and classical aligners stratified by reference variability category. Boxplots show paired differences (BABAPPAlign minus comparator) in SP score, TC score, and runtime within each RV class. Positive score differences indicate higher alignment accuracy for BABAPPAlign; positive runtime differences indicate longer runtime for BABAPPAlign.
